# Low-level laser therapy for treatment of venous ulcers evaluated with the Nursing Outcome Classification: study protocol for a randomized controlled trial

**DOI:** 10.1186/s13063-018-2729-x

**Published:** 2018-07-12

**Authors:** Taline Bavaresco, Ananda Ughini Bertoldo Pires, Vítor Monteiro Moraes, Viviane Maria Osmarin, Denise Tolfo Silveira, Amália de Fátima Lucena

**Affiliations:** 1 Nursing School at Universidade Federal do Rio Grande do Sul, São Manoel, 963, Rio Branco, Porto Alegre, 90620-110 Brazil; 2 Nursing School at Universidade Federal do Rio Grande do Sul, Hospital de Clínicas de Porto Alegre, São Manoel, 963, Rio Branco, Porto Alegre, 90620-110 Brazil; 3 Hospital de Clínicas de Porto Alegre, Ramiro Barcelos, 2350, Santa Cecilia, Porto Alegre, RS 90035-903 Brazil; 4 Caxias do Sul, Brazil

**Keywords:** Low-level light therapy, Venous ulcer, Outcome assessment (health care), Randomized controlled trial, Nursing care, Nursing assessment, Laser therapy

## Abstract

**Background:**

Different methods are available for the treatment of venous ulcers. Most current approaches focus on a combination of topical and compressive therapy. Adjuvant low-level laser therapy may be helpful in lesions with a protracted healing course, but evidence for its use is still limited. This paper describes the protocol of a randomized controlled trial designed to compare the effect of adjuvant low-level laser therapy versus conventional venous ulcer tissue repair, evaluated by a nurse using clinical indicators from the Nursing Outcomes Classification (NOC).

**Methods/design:**

For this prospective randomized controlled trial, 40 adult patients of both sexes with active venous ulcers will be recruited. Subjects will be selected by the sealed-envelope method without any annotation or external identification that might refer to the type of study group. At the time of unblinding, a label with the description of the group to which the patient belongs (that is, control or intervention) will be found inside the envelope. Conventional treatment (topical medication and compressive therapy) will be offered to both groups. Additionally, the intervention group will receive adjuvant low-level laser therapy. All patients will be followed weekly until ulcer healing or for a maximum of 16 weeks. Evaluation of tissue repair will be based on 14 clinical indicators drawn from NOC for wound healing (secondary intention) and tissue integrity (skin and mucous membranes). The primary endpoint will be decreased wound size and scar formation. This laser therapy is expected to enhance the quality, speed, and effectiveness of the treatment of venous ulcers, a chronic condition. This should reduce associated costs to the health service and allow patients to resume their daily activities sooner.

**Discussion:**

This randomized clinical trial will use a validated method to investigate the effect of a novel intervention for the treatment of venous ulcers.

**Trial registration:**

ClinicalTrials.gov, NCT03229330. Registered on July 2017.

**Electronic supplementary material:**

The online version of this article (10.1186/s13063-018-2729-x) contains supplementary material, which is available to authorized users.

## Background

Chronic venous insufficiency can be defined as the set of clinical manifestations caused by reflux and/or obstruction of the peripheral venous system (superficial, deep, or both), usually affecting the lower limbs [1]. Clinical examination and a taking thorough history, which usually reveal clinical manifestations attributed to venous involvement (such as tingling, pain, burning, muscle cramps, edema, pruritus, restless legs, and fatigue), are the first steps in diagnosis. Diagnostic accuracy can be enhanced with Doppler ultrasonography, a noninvasive imaging modality that evaluates the anatomy of the venous system and its physiology by a hemodynamic evaluation of blood flow [2]. The consequences of chronic venous insufficiency include edema, hyperpigmentation of the skin, and, often, development of superficial, irregularly shaped venous ulcers.

Venous ulcers represent about 70–90% of all leg ulcers, with a lifetime prevalence of 0.1% to 2% in the world population. Incidence is higher in individuals over 65 years of age, and women are proportionally more affected, due to their higher survival rates compared to men [3, 4]. The natural history of a venous ulcer is a continuous cycle of healing and tissue derangement that can persist for a long time, with substantial morbidity and recurrence in approximately 70% of cases [3, 4]. The negative impact of venous ulcers on quality of life and the high costs associated with their treatment mean there is a pressing need for new therapeutic options. Conventional venous ulcer treatment is currently based on a combination of topical care (wound dressing) and compressive therapy, as well as educating patients on self-care, which includes wound dressing, hygiene, diet, and exercise [5, 6].

Low-level laser therapy (LLLT) has been used as an adjuvant to conventional therapy with promising results, especially in patients with acute and bloody ulcers [7–9].

### Low-level laser therapy

Proper wound care requires dressings that provide an ideal environment for the wound bed and the healing process, with properties such as hydration, thermal insulation, elimination of necrotic tissue, bacterial control, and adequate pH level [7–9]. Thus, the dressing and medication need to be appropriate to the type of tissue injured, so that they can have the intended healing effect. When using a conventional topical medication, numerous factors may interfere with its action on the treated tissue. In this context, conventional care can be augmented with new technologies in an attempt to minimize this interference and potentiate wound healing. LLLT is a promising adjuvant wound-care technology, which may produce additional beneficial effects in tissue regeneration.

LLLT is a form of phototherapy that employs electromagnetic radiation capable of generating enough energy to interact with living tissues. It produces photochemical and photophysical effects without generating heat, with the intention of reestablishing cell homeostasis. Essentially, light energy is delivered topically in a controlled, safe manner and it is absorbed by photo-absorbers (chromophores) that transform it into chemical energy [8]. Positive effects include acceleration of tissue repair, increased formation of granulation tissue, wound contraction, inflammation modulation, and pain reduction [7, 8]. The biochemical effects of LLLT are associated with the release of preformed substances (histamine, serotonin, and bradykinin), which stimulate the production of ATP and inhibit the production of prostaglandins. Moreover, the bioelectric effects of laser light improve the functioning of the sodium–potassium pump (Na^+^/K^+^-ATPase, which is responsible for maintenance of the cell membrane potential), again increasing the production of ATP. The resulting energy is used to normalize cellular and tissue functions according to normal tissue genetics and physiology, thus promoting more consistent tissue repair [7–10].

Although several studies have demonstrated positive outcomes with LLLT, little is known about its beneficial effects on the treatment of chronic wounds such as venous ulcers. A systematic review of four randomized clinical trials in which laser therapy was administered to patients with a diabetic foot ulcer over 2 to 16 weeks found favorable outcomes regarding ulcer size and time to healing [7]. Another randomized clinical study of 51 patients with leprosy wounds reported a clinical improvement with LLLT, although there was no statistically significant difference between the control group (CG) and intervention group (IG) [11].

These trials notwithstanding, evidence is still limited, with most coming from observational studies. The authors of these studies note that more robust research is still needed to enable standardization of parameters and expansion of LLLT to other clinical scenarios, such as venous ulcers. Additional randomized clinical trials are particularly necessary to bridge this knowledge gap [12–16].

### Evaluation of tissue regeneration

The monitoring of tissue regeneration is based on a nurse’s clinical judgment regarding the characteristics of the wound in relation to its bed, its edges, and the perilesional skin. This evaluation guides the choice of wound-care medication to be used, but is susceptible to evaluator error, because there are no standardized clinical indicators.

The evaluation of tissue regeneration can be enhanced using classification systems. Among such systems, the Nursing Outcomes Classification (NOC) is particularly interesting as it provides a list of clinical indicators for evaluating a patient’s condition and the clinical course. These indicators are scored on 5-point Likert scales (range 1 to 5), in which the lowest score represents the worst possible state and the highest score represents the most desirable state after implementation of interventions [9–17]. However, research into the clinical applicability of the NOC in wound evaluation is still incipient, which makes it impossible for nurses to know which clinical indicators to measure during the clinical follow-up of patients with venous ulcers. This randomized clinical trial should provide clinical evidence of the effect of LLLT as an adjuvant treatment for venous ulcers when compared to conventional topical treatment. Intervention effects and clinical evolution will be assessed through a reliable, validated method, namely, the clinical indicators of the NOC.

The aim of this article is to describe the protocol of a randomized controlled trial designed to compare the effect of conventional venous ulcer treatment with adjunctive LLLT versus conventional treatment alone. Throughout, patients will be evaluated with the clinical indicators described in the NOC.

## Methods/design

### Study design and centers

**Fig. 1:**
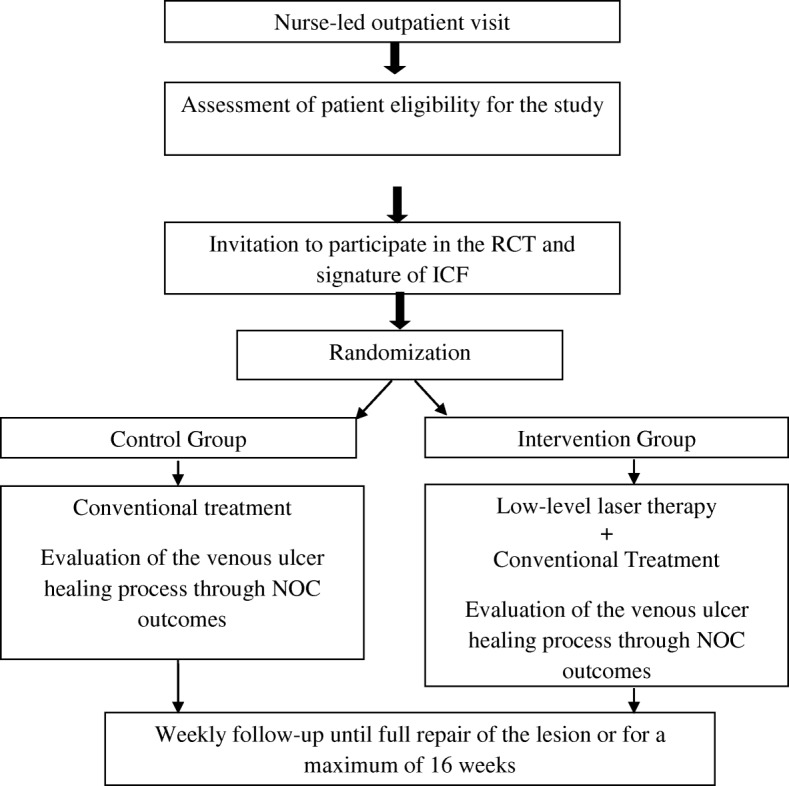
Schedule of enrollment, interventions, and assessments of the low-level laser therapy intervention

This randomized clinical trial will be conducted on a sample of participants recruited from the outpatient clinics of Hospital de Clínicas de Porto Alegre (HCPA), a tertiary-care center affiliated with the Federal University of Rio Grande do Sul, Brazil. At HCPA, patients with venous ulcers receive care from nurses, who perform nursing consultations with a focus on wound dressing and patient education. This study is conducted in accordance with the Standard Protocol Items: Recommendations for Interventional Trials (SPIRIT) Checklist (Additional file 1 and Fig. 1).

### Inclusion and exclusion criteria

The study will include adult subjects (age ≥ 18 years) of both sexes with an established diagnosis of chronic venous insufficiency and an active venous ulcer. All must also be available to attend the outpatient clinic weekly.

Exclusion criteria include a body mass index consistent with grade 3 obesity, current treatment for cancer, erysipelas, cellulitis, lymphangitis, chronic lymphedema, immunosuppressant and/or corticosteroid therapy, a circular venous ulcer, and coagulation necrosis covering more than 25% of the wound bed.

The following exclusion criteria were adopted because the conditions increase the average time to chronic wound healing even further, and would, thus, require a longer follow-up period than specified for the study: circumferential ulcers, due to their large extent; lymphangitis, erysipelas, and cellulitis, because they prolong the inflammatory phase of the wound and, consequently, hinder the tissue repair process; morbid obesity, because it hinders cellular nutrition and can make it difficult for patients to complete the proposed exercises and change their own dressings; and finally, active treatment for cancer because it is an established contraindication to laser therapy.

### Ethical considerations

Participants will be given information about the study and will read and sign an informed consent form before entering the study. Continuity of conventional treatment after completion of the study will be ensured for both groups. After 6 months, all patients will meet with the study team, who will evaluate tissue repair and their lifestyle, and they will receive specific care to prevent recurrent or new ulcers. The study protocol (15–0634) was approved by the institutional review boards of the hospital and the university. The study will be conducted in accordance with the principles of the Declaration of Helsinki and in accordance with Brazilian Ministry of Health guidelines and legislation for research on human subjects.

### Sample size

The WINPEPI program, version 11.43, was used to calculate the sample size. A sample of 34 subjects (*n* = 17 per group) would be able to detect a 1-point difference on a NOC indicator score between group means (laser intervention versus conventional control) as significant. The indicators are scored on 5-point Likert scales (range 1 to 5), in which the lowest score represents the worst possible state and the highest score represents the most desirable state after implementation of interventions. A change in one level, i.e., one point on the Likert scale, characterizes a positive effect of the intervention implemented throughout the treatment study, according to mixed linear models and generalized estimating equations. The standard deviation common to the groups [18], with a statistical power of 80% and significance level of 5% were defined for the study. To account for possible refusals and losses to follow-up, the sample was oversized by 20%. Thus, 40 subjects (*n* = 20 per group) will be recruited.

### Interventions

The IG will receive LLLT using an Inbramed® system, which emits laser light in the red spectrum (wavelength 660 nm and power 30 mW). The application will occur over the center of the injured area in sweep mode, with the laser tip at least 1 cm away from the bleeding area. At the wound edges and at a distance from the lesion, application will be performed in spot mode, with the laser tip leaning against the skin. The laser tip will be covered with a clear, disposable polyvinyl chloride lens to prevent infection and contamination as a result of direct contact with the lesion. The nurse and patient will wear personal protective eyewear rated for laser use. After laser therapy, a conventional dressing will be applied, and self-care guidelines will be given to the patient as clinically indicated.

The CG will receive conventional treatment alone, which constitutes conventional wound dressing and application of topical medication as indicated by the characteristics of the ulcer and perilesional area (including silver or calcium alginate, petrolatum-impregnated gauze, medium-chain triglycerides, papain, hydrogel, or solid petroleum jelly), followed by compressive therapy and patient self-care guidance as clinically indicated.

### Study protocol

**Fig. 2:**
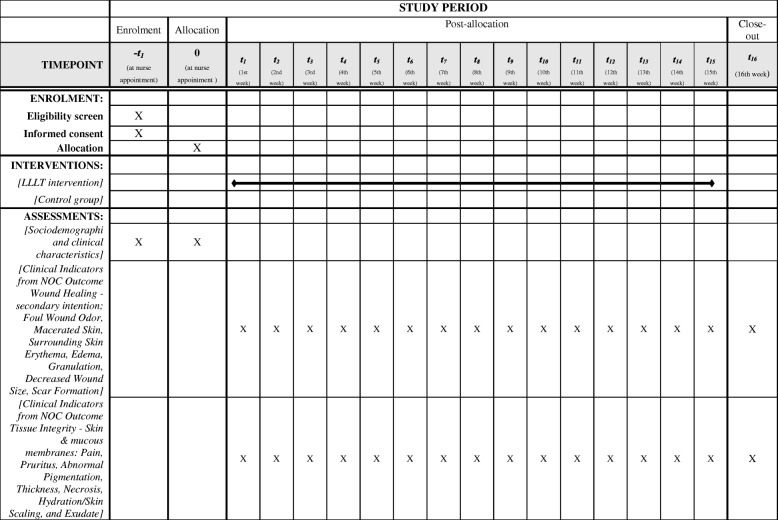
Flow chart of study participation and interventions.ICF, informed consent form, NOC Nursing Outcomes Classification, RCT randomized controlled trial

Patients eligible for the study will be divided into two groups (IG and CG) (Fig. 2). IG patients will receive LLLT as an adjunct to conventional treatment. CG patients will receive conventional treatment alone. After the interventions, patients from both groups will receive guidance on self-care at home.

Conventional treatment in CG participants will be provided by a staff nurse in the outpatient wound-care clinic who has clinical experience in the care of patients with venous ulcers. IG participants will be treated by a nurse who has similar clinical experience in wound care and is also a trained laser therapist. All nurses have been trained in the standard procedures for venous ulcer care and the clinical indicators of NOC outcomes to evaluate these lesions.

In both groups, an evaluation of healing will be carried out by assessing the 14 constituent clinical indicators of the NOC outcomes for wound healing (secondary intention; 1103) and tissue integrity (skin and mucous membranes; 1101). The 14 clinical indicators are foul wound odor, macerated skin, surrounding skin erythema, edema, granulation, decreased wound size, scar formation, pain, pruritus, abnormal pigmentation, thickness, necrosis, hydration/skin scaling, and exudate. Each clinical indicator will be evaluated using a 5-point Likert scale, where the lowest score denotes the worst possible outcome, and the highest score, the best possible outcome.

In both groups, patients will be monitored weekly until complete repair of the ulcer or for a maximum of 16 weeks. The frequency of LLLT application for this protocol was based on previous studies of phototherapy for chronic wounds [12–16]. The duration of treatment was determined based on the average healing period of a chronic wound [7–12].

### Randomization

During nursing consultations at the outpatient clinic, investigators will assess each patient’s eligibility for the study, according to the previously established inclusion and exclusion criteria. Once deemed eligible, the patient will be invited to participate in the study. Those who accept will be asked to sign an informed consent form.

Subsequently, an additional meeting with the research assistant, the patient will be prompted to draw a sealed brown envelope with no external notations or identification that might refer to their group allocation. Inside the envelope, there is a label indicating the group to which the patient belongs (that is, control or intervention). The envelope will then be opened by the patient himself. A research assistant putting the labels inside the envelopes before the study began. It organizes all the instruments that will be necesssary for the application of the research, confers and organizes the consultations agendas.

### Demographic and clinical variables

A structured questionnaire will be administered to all study participants to obtain sociodemographic and clinical information (age, sex, educational attainment, current medications, comorbidities, smoking, alcoholism, chronic venous insufficiency grade, ulcer duration, wound dressing regimen, nutrition, and exercise). Evaluation of the ulcer itself will be performed using the 14 NOC clinical indicators described above. Patients will be instructed to perform daily plantar flexion and extension and calf-strengthening exercises, resting in between, and taught how to dress their wounds as appropriate to their individual needs.

### Outcomes

#### Primary outcome

The primary outcome is decreased wound size and scar formation based on NOC outcome wound healing (secondary intention; 1103).

1. Wound area in cm^2^ (measured as the product of the longest dimension in the cephalocaudal direction by the widest dimension), evaluated by a Likert scale, with 1 being the worst possible score and 5 the best possible score. Each Likert scale score of the Decreased wound size indicator has an operational definition that corresponds to the size in cm^2^.
2. Wound covered with epithelial tissue (new pink or bright tissue that develops from the wound edges or as islands on the surface of the wound), evaluated by a Likert scale, with 1 being the worst possible score and 5 the best possible score.

#### Secondary outcomes

1. Thickness

NOC tissue integrity (skin and mucous membranes; 1101) – depth reached: The layers and structures of the skin altered by loss of tissue integrity (ulcerated area) will be evaluated by a Likert scale, with 1 being the worst possible score and 5 the best possible score.

2. Pain

NOC tissue integrity (skin and mucous membranes; 1101) – Unpleasant sensory and emotional experience arising from actual or potential tissue damage or described in terms of such damage, with sudden or slow onset of mild to severe intensity, constant or recurrent, without an anticipated or predictable termination: The frequency, condition, and intensity will be evaluated by a Likert scale, with 1 being the worst possible score and 5 the best possible score.

3. Overall improvement of other correlated NOC indicators

Overall improvement of the indicators for the NOC outcomes for wound healing (secondary intention; 1103) and tissue integrity (skin and mucous membranes; 1101) will also be assessed.

### Independent/exposure and confounding variables

The main exposure variable will be the tissue repair process in the CG and IG. The impact of each management strategy on the outcomes will be controlled by monitoring the following confounding variables: pharmacotherapy or co-intervention at another health facility or by another professional.

### Statistical analyses

Continuous variables will be expressed as mean and standard deviation or median and interquartile range according to the data distribution. Categorical variables will be expressed as absolute and relative frequencies. Generalized estimating equations will be used for comparisons between the weekly indicators, and the least-significance-difference post-hoc test will be used to assess the difference between weeks.

For quantitative variables with a normal distribution, the difference between the two groups will be compared by Student’s *t*-test. This test will also be used to analyze the means of the NOC results in relation to the decreased wound size and scar formation outcomes. The Pearson correlation coefficient will be used to evaluate the linear association between NOC results and primary outcomes. A nonparametric Mann–Whitney test will be used for comparison between the two groups regarding the characterization of ulcers.

To assess the effect size of the intervention, relative risks with 95% confidence intervals will be calculated. A two-tailed *P* < 0.05 will be considered statistically significant. All analyses will be performed in PASW Statistics, Version 18.0.

## Discussion

The proposed randomized controlled trial will evaluate the efficacy of venous ulcer treatment with low-power laser therapy. Our study is innovative in many ways. We have developed an outpatient wound-care protocol designed to promote faster tissue regeneration with less costly care, combining technology and direct supervision.

As in all studies, we anticipate that there will be potential problems. Recruitment for clinical trials is challenging, as changes in infrastructure and supplies may change during the study. Another point to be discussed is the long duration of follow-up, in this case up to 16 weeks, which may be influenced by other components. The 16-week period being proposed is understood to be adequate, considering the average healing time of a venous ulcer. One limitation is that more complex patients were excluded, due to factors that could prolong healing time. However, our team’s experience in research projects and its ability to measure the outcomes of this intervention are some of our key advantages.

### Trial status

Enrollment is ongoing. Recruitment started in March 2017 and is expected to conclude in December 2018. The first block of randomized patients is already receiving the study interventions, and more participants are being recruited. Target enrollment for the study is 40 subjects.

## Additional file

Additional file 1: SPIRIT 2013 checklist: recommended items to address in a clinical trial protocol and related documents. (DOC 121 kb)

## Abbreviations

CG: Control group
HCPA: Hospital de Clínicas de Porto Alegre
ICF: Informed consent form
IG: Intervention group
LLLT: Low-level laser therapy
NOC: Nursing Outcomes Classification
RCT: Randomized controlled trial
